# Liver Protective Effect of Fenofibrate in NASH/NAFLD Animal Models

**DOI:** 10.1155/2022/5805398

**Published:** 2022-06-17

**Authors:** Ali Mahmoudi, Seyed Adel Moallem, Thomas P. Johnston, Amirhossein Sahebkar

**Affiliations:** ^1^Student Research Committee, Faculty of Medicine, Mashhad University of Medical Sciences, Mashhad, Iran; ^2^Department of Medical Biotechnology and Nanotechnology, Faculty of Medicine, Mashhad University of Medical Sciences, Iran; ^3^Department of Pharmacology and Toxicology, College of Pharmacy, Al-Zahraa University for Women, Karbala, Iraq; ^4^Department of Pharmacodynamics and Toxicology, School of Pharmacy, Mashhad University of Medical Sciences, Mashhad, Iran; ^5^Division of Pharmacology and Pharmaceutical Sciences, School of Pharmacy, University of Missouri-Kansas City, Kansas City, Missouri, USA; ^6^Applied Biomedical Research Center, Mashhad University of Medical Sciences, Mashhad, Iran; ^7^Biotechnology Research Center, Pharmaceutical Technology Institute, Mashhad University of Medical Sciences, Mashhad, Iran; ^8^Department of Biotechnology, School of Pharmacy, Mashhad University of Medical Sciences, Mashhad, Iran

## Abstract

Nonalcoholic fatty liver disease (NAFLD) is initiated by excessive fat buildup in the liver, affecting around 35% of the world population. Various circumstances contribute to the initiation and progression of NAFLD, and it encompasses a wide range of disorders, from simple steatosis to nonalcoholic steatohepatitis (NASH), cirrhosis, and liver cancer. Although several treatments have been proposed, there is no definitive cure for NAFLD. In recent decades, several medications related to other metabolic disorders have been evaluated in preclinical studies and in clinical trials due to the correlation of NAFLD with other metabolic diseases. Fenofibrate is a fibrate drug approved for dyslipidemia that could be used for modulation of hepatic fat accumulation, targeting peroxisome proliferator-activator receptors, and de novo lipogenesis. This drug offers potential therapeutic efficacy for NAFLD due to its capacity to decrease the accumulation of hepatic lipids, as well as its antioxidant, anti-inflammatory, and antifibrotic properties. To better elucidate the pathophysiological processes underlying NAFLD, as well as to test therapeutic agents/interventions, experimental animal models have been extensively used. In this article, we first reviewed experimental animal models that have been used to evaluate the protective effects of fenofibrate on NAFLD/NASH. Next, we investigated the impact of fenofibrate on the hepatic microcirculation in NAFLD and then summarized the beneficial effects of fenofibrate, as compared to other drugs, for the treatment of NAFLD. Lastly, we discuss possible adverse side effects of fenofibrate on the liver.

## 1. Introduction

Hepatic steatosis is caused by an excess buildup of fat in the liver. Various circumstances contribute to the initiation and progression of hepatic steatosis. Nonalcoholic fatty liver disease (NAFLD) is a term that refers to hepatic steatosis caused by unknown factors, and it encompasses a wide range of disorders, from simple steatosis to nonalcoholic steatohepatitis (NASH), cirrhosis, and liver cancer [1–5].

Over the last two decades, nonalcoholic fatty liver disease (NAFLD) has attracted much attention. This metabolic disorder, caused by an accumulation of fat deposits in hepatocytes, affects around 35% of the world population [6]. Several treatments have been evaluated, such as dietary restrictions, insulin sensitizers, and weight reduction, but there is no definitive cure for NAFLD. Although most of these therapies can reduce indicators of liver damage, they cannot ameliorate histological abnormalities [7]. Currently, several medications related to other metabolic disorders are being evaluated in preclinical studies and clinical trials due to the possible correlation of NAFLD with other metabolic diseases such as hyperlipidemia, obesity, hypertension, cardiovascular disease, and insulin resistance [8–10]. For example, PPAR-*γ* agonists, such as pioglitazone, which have been approved to treat type 2 diabetes, act by decreasing endogenous glucose production (EGP) and gluconeogenesis [11]. Pioglitazone also ameliorates hepatic steatosis, peripheral and hepatic inflammation, steatohepatitis, and fibrosis [12]. Additionally, the pharmacological goal of newer insulin sensitizers is to preferentially target the mitochondrial pyruvate carrier, while minimizing direct binding to the transcriptional factor PPAR-*γ* [13]. Importantly, mitochondrial pyruvate metabolism is critical for gluconeogenesis from pyruvate, and consequently, the development of NAFLD. Other metabolic-related drugs are glucagon-like peptide-1 receptor (GLP-1R) agonists, which have been approved to treat obesity and type 2 diabetes. Several investigations have suggested that GLP-1R agonists may represent an alternative strategy for the management of NAFLD by significantly mediating a reduction in insulin resistance, blood glucose, and hepatic lipid content, as well as improving liver histology [14, 15]. However, based on several previous studies, the relation of NAFLD to these metabolic disorders is complex [14, 16, 17]. One of the key drug targeting strategies is the modulation of hepatic fat accumulation, including targeting peroxisome proliferator-activator receptors and de novo lipogenesis [18]. Fenofibrate is a fibrate drug approved for dyslipidemia and is one of the drugs being evaluated for the treatment of NAFLD [19]. Fenofibrate is a PPAR-*α* agonist, which is primarily expressed in the liver. This drug induces oxidation of hepatic lipids, which causes a reduction in the concentration of hepatic triacylglycerol [20]. Specifically, fenofibrate controls lipid and carbohydrate metabolism, promotes lipoprotein lipase activity, and significantly decreases triglyceride levels in the blood. Furthermore, it improves lipoprotein remnant clearance, significantly lowers low-density lipoprotein (LDL) synthesis, and modestly raises high-density lipoprotein (HDL) levels [21, 22]. The mechanism of action for this drug is its agonist effect on peroxisome proliferator-activated receptor (PPAR)*α*. PPAR*α* is abundantly expressed in the liver and modulates various genes involved with the catabolism of fatty acids [23, 24]. It has also been suggested that fenofibrate, which modulates a wide range of important genes involved in NAFLD/NASH, could potentially regulate disease progression [25]. Numerous studies have reported that fenofibrate exhibits potential therapeutic efficacy for NAFLD due to several properties, which include antioxidant, apoptotic (as it relates to cancer cells), anti-inflammatory, and antifibrotic activity [26–29]. Furthermore, several clinical-based research studies have concluded that fenofibrate might have a therapeutic benefit in treating NAFLD/NASH. We summarize these investigations in Table 1 [30–32].

As mentioned previously, experimental animal models have been extensively used to better elucidate the pathophysiological processes underlying NAFLD, as well as to test therapeutic agents/interventions that might potentially be effective for the treatment of fatty liver disease. In this article, we first reviewed experimental animal models that have been used to evaluate the protective effects of fenofibrate on NAFLD/NASH. Next, we investigated the impact of fenofibrate on the hepatic microcirculation in NAFLD and then summarized the beneficial effects of fenofibrate, as compared to other drugs, for the treatment of NAFLD. Lastly, we discuss possible adverse side effects of fenofibrate on the liver.

## 2. In Vivo Impact of Fenofibrate on NAFLD/NASH

While no animal model completely recapitulates the phenotypic signatures of NAFLD/NASH, nevertheless, the commonly used animal models do replicate a number of the most important biochemical and physiological alterations observed with NAFLD/NASH. The most common methods used to induce NAFLD/NASH in experimental animal models include either the administration of dicyclohexylcarbodiimide (DDC) or carbon tetrachloride (CCl_4_) or the use of specialized diets such as the methionine-choline-deficient (MCD) diet, high-fat diet (HFD), high-fat-high-fructose diet (HFFD), high-cholesterol diet (HCD), fructose-enriched diet (FED), choline-deficient L-amino acid-defined (CDAA) diet, and the maternal high-fat diet (mHFD).

**Figure 1:**
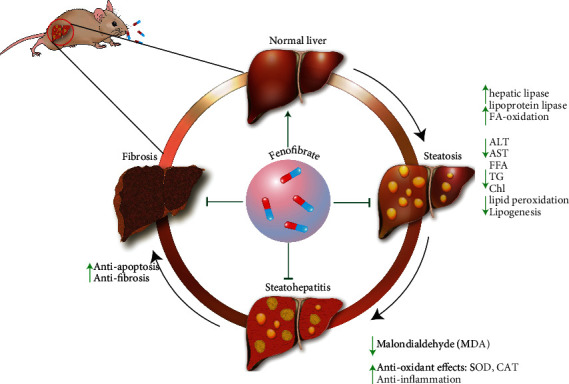
The effects of fenofibrate on NAFLD, which involves several different mechanisms to reduce hepatic lipid accumulation and increase antioxidant, anti-inflammatory, and antifibrotic activity.

Prior to summarizing and discussing the most important and relevant in vivo models of NAFLD/NASH in which fenofibrate has been evaluated, we first present the impact of fenofibrate at different stages in the progression of NAFLD (Figure 1).

## 3. NAFLD/NASH Animal Model Resulting from Ingestion of a High-Fat Diet (HFD)

The HFD model represents one of the standard animal models used to study NAFLD. A HFD typically has a fat content ranging from 45 to 75 energy percent fat [35]. Triglyceride accumulation in the liver results from an excess supply of free fatty acids, which occurs both directly (via ingestion of the HFD) and indirectly (e.g., through enhanced lipolysis) [36]. In this model, steatosis normally occurs after 1-2 weeks, and NASH frequently appears after 12 weeks. As an aside, the steatosis and inflammation that arise upon consumption of a HFD are typically less severe than that observed in animals fed the methionine choline-deficient (MCD) diet, which will be discussed in a later section [37]. It is important to note that animals ingesting a HFD typically exhibit obesity, hyperlipidemia, and hyperinsulinemia after ten weeks, as well as glucose intolerance after 12 weeks. At 34-36 weeks, it has been reported that AST and ALT levels are markedly elevated in animals consuming a HFD [38]. Nevertheless, following prolonged consumption of the HFD, animals are observed to have minimal liver fibrosis [38].

Research in 2015 by Zhang et al. indicated that 20 mg/kg fenofibrate administered to rats maintained on a high-fat and high-sucrose diet elevated fatty acids in the serum, while reducing the fatty acid content in the liver [39]. These authors demonstrated that fenofibrate ameliorated inflammatory cell infiltration and hepatic steatosis. Additional research has revealed that fenofibrate exerts notable antioxidant effects [40]. A different study on apolipoprotein E-2 (APOE2) knock-in mice maintained on a HFD-diet showed that treatment with fenofibrate reduced the accumulation of hepatic macrophages and eliminated steatosis, which is in line with fenofibrate's capacity to downregulate two primary gene groups involved with hepatic lipid turnover and inflammation. These investigators determined that the downregulation of inflammatory genes occurred immediately after fenofibrate administration [41].

Recently, a study using female ovariectomized (OVX) mice fed a HFD showed that six weeks of fenofibrate treatment had significant effects on weight loss (-38%) and adipose tissue mass (-46%). They also reported that several enzymes (ALT and AST), as well as FFA, TG, and total cholesterol, decreased. These findings demonstrated the notable effects of fenofibrate in ameliorating liver damage and dyslipidemia in OVX-mice [42]. Cholesterol metabolism is one vital component in a variety of processes that can cause hepatic inflammation. In fact, hepatic inflammation is a critical factor in the progression of NAFLD to NASH and ultimate liver injury, which can be diminished by fenofibrate [43]. Similar to previous literature reports using various animal models of NAFLD (e.g., LDL receptor-deficient mouse, Otsuka Long-Evans Tokushima fatty rats, mice fed a MCD diet, and mice fed a HFD), this study also demonstrated a decrease in accumulated hepatic lipid (-69%), together with a reduction in macrophage infiltration (-76%) [41, 44–46]. Another study using rats fed a HFD showed that adding folic acid to fenofibrate could improve its effectiveness in controlling the progression of NAFLD. This study also revealed that fenofibrate promotes the production of homocysteine (Hcy), which attenuates the beneficial effects of fenofibrate in treating NAFLD [47]. However, adding folic acid to fenofibrate significantly reduces Hcy levels, possibly due to an increase in its metabolism. Specifically, folic acid mediates Hcy remethylation to methionine [47, 48]. One particular study with Sprague-Dawley rats fed a HFD showed that fenofibrate might reduce hepatic steatosis *via* a process connected to a reduction in hepatocyte apoptosis [49]. Apoptosis has been shown to play a critical role in the progress of NAFLD. It is thought that the role of apoptosis may be mediated through Blc-2 proteins, caspases, and c-Jun N-terminal kinase [50]. Moreover, fenofibrate actively stimulates PPAR*α*, which confers antiapoptotic and anti-inflammatory effects on hepatocytes [51, 52]. It has also been shown that fenofibrate significantly reduces the protein levels of TNF*α*. Additionally, it has also been reported that fat accumulation in Kupffer cells in mice fed a HCD decreased with fenofibrate administration [52].

## 4. NAFLD/NASH Animal Model Resulting from Ingestion of a Fructose-Enriched Diet (FED)

The fructose-enriched diet (FED) induces lipid synthesis and storage and reduces beta-oxidation in the liver [53]. Fructose is a monosaccharide processed predominantly by the liver [54]. Excessive consumption of fructose has been associated with the development and worsening of NAFLD as a result of fat accumulation, oxidative stress, inflammation, insulin resistance, and possibly fibrosis [55]. After eight weeks, consumption of a FED in mice and rats can cause simple steatosis, with no signs of steatohepatitis; although, there is a considerable increase in body weight, glucose levels, and plasma lipids [56, 57]. Despite a significant difference in weight gain between glucose and sucrose consumption in mice and rats, ingestion of a FED produces greater hepatic fat storage than either sucrose or glucose [58].

The effect of fenofibrate administration has been investigated in several fructose-induced NAFLD animal models. Recently, it was reported that inhibition in macrovesicular steatosis was observed with fenofibrate treatment in Sprague-Dawley rats that consumed a FED [59]. In another similar model induced with high dietary fructose, the results demonstrated that fenofibrate inhibited fat accumulation in the liver, reduced oxidative stress, and improved both body weight and glycemic status, which confirmed the antidiabetic and lipotropic properties of fenofibrate [60]. It is worth mentioning that a reduction in body weight may be a consequence of the catabolism of hepatic lipids [61]. It should be noted, however, that other research has demonstrated that fenofibrate does not affect body weight [62]. In a new strategy that involved cotreatment with fenofibrate and antisense oligonucleotide-fat specific protein 27 (ASO-Fsp27) to mice fed a high-trans-fat, high-cholesterol, and high-fructose diet for eight weeks, the results showed improvement in several symptoms of NASH/NAFLD, which included inflammation, hepatic steatosis, fibrosis, and oxidative stress. Importantly, silencing Fsp27 with ASOs alone did not produce these same beneficial effects [63]. Fsp-27 is a fat-specific protein known as lipid droplet-associated protein and modulates triglyceride (TG) storage in hepatocytes [64]. TG storage is hepatocytes that is necessary during fasting [65]. Several investigations in animals and humans have indicated that the expression of Fsp-27 in the liver is profoundly elevated under pathological conditions (e.g., steatosis) [66–69]. The above findings with Fsp27 and fenofibrate would seem to confirm their synergistic effect, as well as highlight the crucial role of fenofibrate, in ameliorating NAFLD and NASH [63].

## 5. NAFLD/NASH Animal Models induced by the Methionine Choline-Deficient (MCD) Diet

Nutrient-deficient diets have been used to explain the inadequate steatohepatitis reaction to most hypercaloric diets in terms of supplying a “second hit” to liver metabolism. Several critical chemical compounds, such as methionine (an essential amino acid with a methyl donor), are either insufficient or absent in nutrient-deficient diets. Additionally, nutrient-deficient diets can be made even less lipotropic by substituting L-amino acids for dietary proteins. Diverse diet types, including the methionine-deficient (MD), choline-deficient (CD), and methionine choline-deficient (MCD) diets, have been developed and are extensively utilized in preclinical steatohepatitis studies. The primary benefit of nutrient-deficient diets is that they induce the histological characteristics of NASH (e.g., mild to moderate fibrosis) in a shorter time frame than obesogenic diets [70].

The aforementioned diets all cause steatohepatitis in varying degrees. For example, NASH can be caused by either feeding a MD, or CD, diet alone, but only the MD diet can cause minor hepatocellular damage [71]. On the other hand, MCD mice develop hepatic macrovesicular steatosis and inflammatory cell infiltration after 1-3 weeks and substantial perisinusoidal fibrosis after 5-7 weeks; hence, this mouse model is widely employed to evaluate the effectiveness of specific pharmacological interventions.

A recent study in mice reported that saroglitazar (3 mg/kg) demonstrated greater efficacy with regard to a reduction in inflammation, steatosis, fibrosis, and ballooning when compared to fenofibrate (100 mg/kg) and pioglitazone (25 mg/kg), and that these two drugs only partially reduced inflammation and improved overall liver function [44]. Another investigation in mice fed a MCD diet demonstrated that a fenofibrate-nanoliposomal formulation (20 mg/kg/day for seven days) resulted in an 11.8-fold increase in the plasma concentration of fenofibrate and approximately a 54.7% decrease in hepatic lipid when compared to fenofibrate alone (i.e., unencapsulated fenofibrate). However, with an increase in the dose to 40 mg/kg/day, these same authors showed that the plasma concentration of fenofibrate increased 57.3-fold, and the reduction in hepatic lipid was 35%, again, when compared to unencapsulated fenofibrate alone. Interestingly, the higher dose of nanoliposomal fenofibrate (40 mg/kg/day vs. 20 mg/kg/day) resulted in a smaller reduction of hepatic lipid [72]. Nevertheless, these results would appear to suggest that the nanoliposomal formulation of fenofibrate was able to efficiently treat NAFLD in the MCD mouse model [72].

The use of nanoparticulates for the delivery of fenofibrate has been explored in vitro as well. Specifically, one study attempted to improve the efficacy of fenofibrate to treat NAFLD using fenofibrate encapsulated in biodegradable polyurethane (PU) nanoparticles. The fenofibrate/PU nanoparticles were evaluated in vitro using HepG2 cells, as well as in mice with NAFLD induced by a MCD diet. Fenofibrate/PU nanoparticles exhibited a sustained release profile for fenofibrate in vitro. Importantly, when compared to fenofibrate alone, the fenofibrate/PU nanoparticles not only significantly decreased the TG content in HepG2 cells incubated with oleic acid but also decreased liver TG levels and increased plasma fenofibrate concentrations in mice with NAFLD [73, 74].

## 6. NAFLD/NASH Animal Models Induced by the Administration of 3,5-Diethoxycarbonyl-1,4-Dihydrocollidine (DDC)

A steatohepatitis mouse model has been developed following treatment with 3,5-diethoxycarbonyl-1,4-dihydrocollidine (DDC) for eight weeks. The DDC mouse model of NAFLD/NASH exhibits cholestasis-related periportal fibrosis, lipid peroxidation, and pericellular collagen deposition [75, 76].

Research on the DDC-induced steatohepatitis model in 2018 by Nikam et al. was designed to investigate the impact of fenofibrate. Using the DDC model, these authors demonstrated that fenofibrate treatment resulted in a significant decrease in inflammation and oxidative stress. Moreover, downregulation of PPAR*α* in the DDC model was inhibited with fenofibrate treatment. Specifically, the morphologic aspects of steatohepatitis, such as disruption of hepatocytes, formation of protein aggregates (Mallory-Denk bodies), and cell ballooning, as well as other features, including an elevation in serum AST and damage to mitochondria, were completely ameliorated with fenofibrate treatment [77]. In one particular study, the administration of 80 mg/kg fenofibrate per day to rats with fatty livers resulted in a reduction in TG serum levels but no change in serum cholesterol levels. These authors also determined that hepatic lipase (HL) and lipoprotein lipase (LPL) activity increased in both the serum and liver, while levels of malondialdehyde (MDA) were reduced. Furthermore, their findings demonstrated that fenofibrate administration to rats with fatty liver resulted in a significant decrease in the fat content of liver tissue, as well as amelioration of the pathological changes normally observed with fatty liver [78].

## 7. Hereditary Fatty Liver Shionogi (FLS) NAFLD/NASH Animal Model

Shionogi & Co. (Shiga, Japan) developed polygenetic fatty liver Shionogi (FLS) lean animals, which develop spontaneous insulin resistance, steatohepatitis, and hypertriglyceridemia under typical environmental circumstances [79]. In this model, hepatic fibrosis is mild [80, 81], and, to date, only lipid-lowering drugs have been investigated in this model. A hybrid genetic variation of the ob/ob mouse model by backcross mating FLS-ob/ob animals at Tottori University (Yonago, Japan) has created a more robust fibrotic NASH model. This model produces obesity, severe hepatic steatosis, diabetes, necroinflammation, age-dependent development of pericellular fibrosis, and (to some extent) spontaneous carcinogenesis due to combining the traits of both genetic models [81]. Those two introduced models are prevalently used to characterize putative anti-NASH drugs.

Other research pertaining to the use of fenofibrate in animal models includes the use of the hereditary fatty liver Shionogi (FLS) mouse model. Using this particular model, 0.1% fenofibrate treatment for 12 days demonstrated a reduction in hepatic lipid peroxidation, which was suggested to potentially result from mitigation of steatosis. These authors also determined that there was an increase in the protein expression of two critical antioxidant enzymes, including catalase and Cu/Zn-superoxide dismutases (SOD); although, there was no significant increase in the activity of the SODs. It was also reported that a significant increase in catalase activity, as a result of fenofibrate treatment, significantly reduced lipid peroxidation [82].

## 8. In Vitro NASH/NAFLD Models

In vitro models of NASH/NAFLD typically use palmitic acids, or oleic acid, to induce fat accumulation in hepatocellular cell lines such as HepG2, HepaRG, and LX-2. The effects of fenofibrate in a variety of in vitro models of fatty liver have been investigated. Although only a few studies using in vitro models to investigate NAFLD could be found in the literature, these in vitro models are essential for the investigation of other liver disorders. Immortalized cell lines and primary cell cultures are commonly utilized to establish in vitro experimental models of fatty liver disease. Primary human hepatocytes, stellate cells, Kupffer cells, or sinusoidal endothelial cells have been reported to be the appropriate cells for these in vitro models [83, 84]. However, it should be noted that there are ethical concerns and sampling restrictions pertaining to the use of these cell cultures. An alternative to primary cell cultures are immortalized cell lines, which have a prolonged replicative ability, a stable phenotype, and result in consistent cell cultures. Furthermore, these cell line cultures are more convenient and easy to use [85].

In an in vitro model of NASH/NAFLD with HepaRG cells, which was established by exposure to supraphysiologic concentrations of oleic acid, it was found that intracellular TG accumulation was reduced by up to 50% after treatment with fenofibrate. The reduction in intracellular TG was suggested to be the cause for induction of fatty acid oxidation following exposure of the cells to fenofibrate over a 2-week period [86]. Moreover, in a recent study with several in vitro cell models of NASH/NAFLD, including HepG2, HepaRG, and hepatic cells obtained from the skin of human stem cell (hSKP-HPC), primary human hepatocytes (PHH), and LX-2 cells, numerous PPAR*α* agonists, such as fenofibrate, showed a decrease in the induction of these cell culture-based models of NASH [87].

An in vitro study using a coculture of HepG2 and HepG2-LX2 cells showed that three drugs, including pioglitazone, saroglitazar, and fenofibrate, at a concentration of 10 *μ*M, could reverse palmitic acid-mediated changes in mitochondrial dysfunction, NFkB-phosphorylation, ATP production, and stellate cell activation [44].

Therefore, based on the above discussion of animal and in vitro cell-based models of NASH/NAFLD, it would appear that fenofibrate may be potentially beneficial in the prevention and treatment of fatty liver disease.

## 9. Effect of Fenofibrate on the Hepatic Microcirculation in NAFLD

Hepatic veins and portal venous systems are valveless veins that have a critical role in blood circulation. The blood in the liver is typically obtained by tributaries and circulated among the hepatic sinusoids [88]. Excess fatty acids derived from either lipolysis or the diet are released into this network of sinusoids, which triggers the development of NAFLD [89, 90]. The reduction of blood flow in the hepatic microcirculation results in impaired transfer of blood between the microvasculature and extrahepatic blood in the general systemic circulation. Consequently, there is insufficient oxygen and nutrients needed for oxidizing fatty acids in hepatocytes, which stimulates the development of more severe NAFLD [91]. Fat accumulation in the liver can also affect the hepatic microcirculation by decreasing the number and volume of functional sinusoids and decrease hepatic blood flow [92, 93]. One particular earlier study investigated the effects of fenofibrate on hepatic microcirculation in a high-fat diet- (HFD-) induced fatty liver in mice. In vivo imaging analysis revealed the adverse effects of HFD on hepatic vasculature with narrowing of hepatic sinusoids and hepatic microcirculatory perfusion. These same authors also found that oxygen tension was significantly decreased in portal venules, while NADH autofluorescence in hepatocytes was greatly elevated. It was concluded that fenofibrate treatment significantly improved microvascular patency, tissue oxygenation, and redox states in the affected liver and played a beneficial role in terms of regulating both lipid metabolism and the microvascular environment [94]. It is worth noting that NADH and oxygen are two important factors for achieving a balance between TG synthesis and fatty acid oxidation in the liver. By affecting this balance, fenofibrate may inhibit the progression of steatosis in the liver [94]. Finally, fenofibrate has been previously demonstrated to have a vasoprotective influence. It performs this function by promoting nitric oxide production and suppressing the production of endothelin-1 (ET-1). Thus, genes that are involved with sinusoidal microcirculation and vascular tone could potentially be affected following the administration of fenofibrate [95, 96]. In summary, based on the above studies, it would appear that fenofibrate ameliorates NAFLD through a variety of mechanisms.

## 10. Effect of Fenofibrate on NAFLD in Comparison to Other Drugs

Various drugs and natural products are being studied to treat fatty liver diseases, such as pioglitazone, ezetimibe, niacin, Xuezhikang, saroglitazar, gemfibrozil, resveratrol, and rosuvastatin [97–101]. Some of these drugs have shown beneficial effects in terms of improving NAFLD. In this section, we compare the therapeutic effects of fenofibrate to other agents (both conventional, synthetic drug substance, as well as natural products) in treating NAFLD.

We begin our discussion with a randomized clinical trial (*n* = 90) designed to compare fenofibrate with pioglitazone (a PPAR*γ* agonist) for treatment of NAFLD. This trial showed that there was no significant difference in all initial clinical parameters between subjects that received either fenofibrate or pioglitazone. However, following completion of both drug treatments, measurements of systolic and diastolic blood pressure, aspartate transaminase (AST) and alanine aminotransferase (ALT) activity, and blood lipids (as well as blood glucose), were all reduced; although, there was a greater decrease in the activity of both AST and ALT with pioglitazone than with fenofibrate [7]. This study showed that both fenofibrate and pioglitazone provide beneficial effects in patients with fatty liver.

We now consider another drug (saroglitazar; PPAR*γ* agonist) for its effects on NAFLD. An in vitro study using a coculture of HepG2 and HepG2-LX2 cells showed that three drugs, including pioglitazone, saroglitazar, and fenofibrate, at a concentration of 10 *μ*M, could reverse palmitic acid-mediated changes in mitochondrial dysfunction, NFkB-phosphorylation, ATP production, and stellate cell activation. These authors reported that in the in vivo portion of their study using saroglitazar (3 mg/kg) demonstrated greater efficacy with regard to a reduction in inflammation, steatosis, fibrosis, and ballooning when compared to fenofibrate (100 mg/kg) and pioglitazone (25 mg/kg), and that these two drugs only partially reduced inflammation and improved overall liver function [44]. A different study by van Der Veen et al. revealed that phosphatidylethanolamine *N*-methyltransferase- (PEMT-) deficient mice fed a HFD are completely protected from developing insulin resistance and obesity. Nevertheless, they also showed severe progression of NAFLD with this mouse model. These same investigators compared two drugs, including fenofibrate and ezetimibe, in an effort to prevent the development of NAFLD in the mice. Their results showed that fenofibrate significantly reduced fat accumulation, inflammation, and markers of hepatic fibrosis in the PEMT^−/−^ mice, together with maintaining a protective effect from obesity and insulin resistance as mentioned directly above. However, in contrast to fenofibrate, ezetimibe did not demonstrate improvement in these parameters [102]. Importantly, fenofibrate partially reversed hepatic steatosis and fibrosis in *PEMT^−/−^* mice when treatment was initiated even after NAFLD had been established. In summary, their work concluded that increasing hepatic fatty acid oxidation via fenofibrate reversed fatty liver disease in mice lacking PEMT [102]. In another study, fenofibrate was compared with Xuezhikang. Xuezhikang is a traditional Chinese natural product containing cholestin components that can inhibit 3-hydroxy-3-methylgutaryl coenzyme A reductase [103]. In various NAFLD rat models, oral administration of fenofibrate at a dose of 100 mg/kg, or Xuezhikang at a dose of 300 mg/kg, for 42 days showed a decrease in the accumulation of liver fat and ameliorated oxidative stress. It should be noted that insulin resistance usually occurs with NAFLD and exacerbates hepatic steatosis and liver fibrosis, but both fenofibrate and Xuezhikang lessened these adverse effects of NAFLD. Additionally, collagen deposition and necroinflammation decreased after treatment. These authors did not demonstrate a significant therapeutic difference between these two drugs as it pertains to NAFLD; although, they did suggest that the side effects of fenofibrate require further investigation before fenofibrate gains acceptance as an approved therapy for NAFLD [104].

In other investigations, fenofibrate has been compared with niacin. Both fenofibrate and niacin lower plasma TG in obese NAFLD patients without disturbing intrahepatic TG content, because the decrease in plasma and liver TG occur by different mechanisms. Specifically, secretion of VLDL-TG by the liver is reduced with niacin, whereas the plasma clearance of VLDL-TG is increased with fenofibrate. However, one adverse side effect observed with niacin is a decrease in insulin sensitivity in skeletal muscle, liver, and adipose tissue, which does not occur with fenofibrate [34]. Other drug comparisons for NAFLD in rat models have been conducted by Abuja et al. using the hypolipidemic drug gemfibrozil. Fenofibrate improved various parameters including the GFR and plasma homocysteine levels, whereas this was not observed with gemfibrozil (a less potent PPAR*α* agonist) treatment [47]. Other research has shown that using half the dose of fenofibrate (as a PPAR*α* agonist) and half the dose of pioglitazone (as a PPAR*γ* agonist) had greater efficacy than the dose of either drug alone, which was suggested to be due to better modulation and balance in the expression of several adipose and hepatic tissue genes when a combination of PPAR*α* and PPAR*γ* agonists is used together [105]. It has also been reported that a lower dose of fenofibrate can be used when it is combined with resveratrol, which could potentially have a protective effect on liver with regard to steatosis than either agent alone [106]. Resveratrol is a polyphenolic natural product with anti-inflammatory, antioxidant, and antihyperlipidemic properties [107]. Another study using the STAM mouse model showed that a combination of fenofibrate and rosuvastatin could reduce TG levels in the plasm without affecting insulin sensitivity. However, when compared to rosuvastatin, fenofibrate more effectively inhibits the development of hepatic steatosis based on histochemical analyses [62]. Rosuvastatin is a potent inhibitor of HMG-CoA reductase and hepatic cholesterol biosynthesis [108].

## 11. Adverse Side Effects with High-Dose Fenofibrate Treatment

Several reports have indicated that fenofibrate does exhibit some adverse effects. One such study reported that this drug enhanced various genes implicated in fatty acid uptake and transport, *β*-oxidation, and TG synthesis, which enhanced TG content in the liver. These authors reported that fenofibrate modulates TG content in the serum and liver in a dose-dependent manner. They also observed an elevation in the activity of ALT and AST [109]. Nevertheless, several studies have proposed that this hepatic TG accumulation might have a protective role in preventing the toxic effects of free fatty acids [110, 111]. Fenofibrate might also limit further development of hepatic inflammation *via* hepatic stellate cells [112]. It has also been demonstrated that fenofibrate causes weight loss at high doses, but not at low doses [109, 113]. These authors reported that fenofibrate might promote hepatic TG accumulation by increasing the expression of SREBP-1c [109]. Specifically, it has been proposed that the accumulation of TG in the liver as a result of fenofibrate treatment results from direct regulation of PPAR*α* binding to the DR1 motif of SREBP-1c, which results in increased expression of mature SREBP-1c [105, 109]. In humans with obesity, a portal hypothesis has been suggested in which free fatty acids (FFA) and cytokines released from adipose tissue are directly connected to the liver circulation (Figure 1) [114, 115]. Furthermore, fenofibrate, by its agonist effect on PPAR*α*, triggers the secretion of fatty acids by, and lipolysis in, white adipose tissue, which are taken up by the liver and results in lipid accumulation [102, 116, 117]. On the other hand, by inhibiting the secretion of VLDL-TG from the liver, fenofibrate serves to ameliorate hypertriglyceridemia. Raising fatty acid oxidation in hepatic cells can compensate for the lower VLDL-TG secretion rate and cause reversal and/or prevention of NAFLD [102]. Several studies have also reported that high-dose fenofibrate can promote mitochondrial dysfunction and hepatic toxicity [118, 119]. Specifically, these studies have shown that at a daily dosage of 400 mg/kg, fenofibrate treatment caused a reduction of the liver NAD^+^/NADH ratio, induced hyperacetylation of peroxisomal bifunctional enzyme (ECHD) and acyl-CoA oxidase 1 (ACOX1), and induced excessive accumulation of long-chain fatty acids (LCFA) and very-long-chain fatty acids (VLCFA) [118]. More recent research has demonstrated that overall strength was decreased in laboratory animals following oral dosing of 0.3% fenofibrate. This was suggested to result from fenofibrate's genotoxic effect on hepatocyte mitochondrial DNA (mtDNA), which was attributed to an accumulation of ROS. Furthermore, the increase in ROS was thought to occur due to both an increase in peroxisomal *β*-oxidation and reduced expression of genes encoding proteins involved with antioxidant defense. Thus, these authors proposed that fenofibrate affects metabolism of hepatic lipids and is associated with an increase in oxidative stress, which, in turn, mediates oxidative damage to mtDNA [120]. Lastly, a previous study also showed that fenofibrate exacerbated hepatomegaly and aminotransferase abnormalities in the HFD animal model of NAFLD [104, 121].

## 12. Conclusion and Perspectives

This review discussed the possible protective effects of fenofibrate on NAFLD and delved into the diverse mechanisms that lead to a reduction in TG, cholesterol, FFA, lipogenesis, and liver enzymes (e.g., ALT and AST). Fenofibrate not only promotes FA-oxidation but also affects the activity of hepatic lipase and lipoprotein lipase. Lastly, fenofibrate possesses antioxidant and anti-inflammatory properties. In this review, we have presented numerous studies demonstrating that fenofibrate can provide beneficial effects in preventing the progression, or even reversing, the severity, and extent of NAFLD. Based on comparative studies, fenofibrate, in conjunction with a PPAR-*γ* agonist, demonstrated a more significant therapeutic impact on NAFLD than either agent alone. Moreover, fenofibrate has vasoprotective properties, which can potentially help to regulate both the hepatic sinusoidal microcirculation and vascular tone, and thus, ameliorate NAFLD/NASH. Currently, it is suggested that fenofibrate be administered orally at a dose of 200 mg/day in combination with other drugs, specifically PPAR-*γ* agonists. This combination significantly reduces fatty liver in patients with NAFLD. Furthermore, several novel formulations have been developed and suggested for the treatment of NAFLD, such as a simultaneous coassembly of fenofibrate and ketoprofen peptide [122], as well as fenofibrate-containing polyurethane nanoparticles [73] and nanoliposomes [72]. These formulation strategies have been undertaken to both increase hepatocyte targeting and drug efficacy. Other novel formulation approaches, such as the surface engineering of fenofibrate nanocrystals, might also produce feasible drug products for the treatment of NAFLD [123]. However, irrespective of the particular state-of-the-art fenofibrate formulation developed, it is our recommendation that extensive clinical studies must first be conducted to obtain more conclusive and accurate data with regard to the therapeutic efficacy of fenofibrate in the treatment of NAFLD/NASH, especially since, at present, there is no cure for NAFLD/NASH.

## Figures and Tables

**Table 1:** Different human studies of fenofibrate on NAFLD/NASH.

Diseases	Dose	Number of subjects	Duration of the intervention	Effect/side effect	Ref
Hypertriglyceridemia and NAFLD	200 mg	53	12 weeks	(i) Increasing: TLF, LV, plasma acetylcarnitine, butyrylcarnitine levels (ii) Decreasing: serum TG, plasma docosahexaenoic acid	[32]
NAFLD	200 mg fenofibrate per day	90	12 weeks	(i) Decreding: ALT, AST, SBP, DBP, BMI	[7]
NAFLD	300 mg daily	90	24 weeks	(i) No effect on lipid panel (ii) Beneficial effect on indirect biochemical markers of hepatic fibrosis, (hyaluronic acid), TGF-beta 1 (iii) Beneficial effect on inflammatory pathway (iv) Beneficial effect on insulin resistance (v) Beneficial effect on liver stiffness	[30]
NASH	—	124	12 weeks	(i) Improve serum transaminase (ii) Improve clinical symptoms (iii) Improve blood lipids	[33]
Obese-NAFLD	200 mg/day	27	8-16 weeks	(i) Increasing: VLDL-TG clearance of plasma (ii) Decreasing: VLDL-apolipoprotein B secretion (iii) No rise in VLDL-TG secretion (iv) No effect on insulin action	[34]
NAFLD	200 mg/day	17	48 weeks	(i) Increasing: ApoA1 (ii) Decreasing: TG, glucose, ALP, GGT (iii) Without adverse events	[31]

TLF: total liver fat; LV: liver volume; TG: triglyceride; AST: aspartate transaminase; ALT: alanine aminotransferase; VLDL: low-density lipoprotein; ALP: alkaline phosphatase; GGT: *γ*-glutamyltranspeptidase; ApoA1: apolipoprotein A1.

## Data Availability

There is no raw data associated with this review article.
